# *Thymus Citriodorus* (Schreb) Botanical Products as Ecofriendly Nematicides with Bio-Fertilizing Properties

**DOI:** 10.3390/plants9020202

**Published:** 2020-02-06

**Authors:** Nikoletta Ntalli, Athanasia Bratidou Parlapani, Kaliopi Tzani, Maria Samara, George Boutsis, Maria Dimou, Urania Menkissoglu-Spiroudi, Nikolaos Monokrousos

**Affiliations:** 1 Benaki Phytopathological Institute, 8 S. Delta Str., Department of Pesticides Control and Phytopharmacy, 14561 Athens, Greece; bl01481@uoi.gr (K.T.); m.samara@bpi.gr (M.S.); 2 Pesticide Science Laboratory, School of Agriculture, Faculty of Agriculture Forestry and Natural Environment, Aristotle University of Thessaloniki, 54124 Thessaloniki, Greece; mpratidou@agro.auth.gr (A.B.P.); rmenkis@auth.gr (U.M.-S.); 3 Department of Ecology, School of Biology, Faculty of Sciences, Aristotle University, 54124 Thessaloniki, Greece; gboutsis@bio.auth.gr (G.B.); mardimdim@bio.auth.gr (M.D.); 4 Department of Science and Technology, International Hellenic University, 57001 Thessaloniki, Greece; nmonokrousos@ihu.gr

**Keywords:** Lemon thyme, *Meloidogyne* spp., soil microbes, soil free-living nematodes, essential oil, geraniol

## Abstract

In recent years, interest has surged in the development of plant extracts into botanical nematicides as ecofriendly plant protection products. Aromatic plants are maybe the most studied category of botanicals used in this direction and the yielding essential oils are obtained on a commodity scale by hydro distillation. Nevertheless, can the bioactivity of aromatic plants always be attributed to the terpenes content? What would it mean for soil microcosms to bear the treatment of an essential oil to cure against *Meloidogyne* sp.? Are there other extraction procedures to prepare more ecofriendly botanical products starting from an aromatic material? Lemon thyme is studied herein for the first time for its nematicidal potential. We compare the efficacy of lemon thyme powder, macerate, water extract and essential oil to control *Meloidogyne* *incognita* (Chitwood) and *Meloidogyne javanica* (Chitwood), and we additionally study the secondary effects on soil microbes and free-living nematodes, as well as on tomato plant growth. According to our results lemon thyme powder enhances tomato plants’ growth in a dose-response manner and when it is incorporated in soil at 1 g kg^−1^, it exhibits nematicidal activity at a 95% level on *M.* *incognita*. The water extract yielding from the same dose is nematicidal only if it is left unfiltered; otherwise only a paralysis effect is demonstrated but inside the soil the biological cycle of the pest is not arrested. The essential oil is good both in performing paralysis and biological cycle arrest, but it detrimentally lowers abundances of bacterial and fungal feeding nematodes. On the contrary, lemon thyme powder and unfiltered water extract augments the bacterial biomass, while the latter also increases the bacterivorous nematodes. Overall, the bio fertilizing lemon thyme powder and its unfiltered water extract successfully control root knot nematodes and are beneficial to soil microbes and saprophytic nematodes.

## 1. Introduction

In the recent years, a global trend has been employed to develop ecofriendly plant protection products (PPPs) so as to ascertain environmental sustainability and human health [1,2]. In the frame of developing natural PPPs, the insecticidal action of plant essential oils (EOs) has been an area of intensive research. Although the commercialization of EOs has lagged far behind in the past, they have been available in the USA for over a decade, and over the last 4–5 years in the EU [3]. Appropriate technologies have been studied for the utilization of botanical insecticidal preparations, from the “do-it-yourself” smallholder farmer, to a cottage- or village-level collective, to private-sector industry [4]. The mechanism of action of some botanical PPPs has been delineated [3,5], together with the management of resistance development through the detoxification of enzyme activities [6]. Lemon thyme (Lamiaceae) is a lemon-scented evergreen mat-forming perennial. In Greece, it is cultivated mainly for culinary purposes. *Thymus citriodorus* (Schreb) is reported to exhibit biological activities like antioxidant and inhibitory effects against aflatoxin-producing *Aspergillus flavus* (Link) fungus [7], antimicrobial activity against foodborne bacteria [8] and cytoprotective effects [9]. It has even been investigated for its pleasant hint of lemon fragrance [10], for how cultivation practices affect its yields in EOs [11], along with the best phenological stage for harvest [12,13,14], and extraction ways to ameliorate its yield in EOs [15] and its main bioactive component, thymol [16].

To the best of our knowledge, nothing is known on the activity of lemon thyme against parasites and pathogens of agronomic interest and especially root knot nematodes. Of course, many other botanicals have been studied for the nematicidal properties of their yielding EOs [17,18,19], while nanotechnology has made progress in the introduction of EOs into open field conditions [20,21,22]. However, there are few studies on the ecotoxicological effects of biopesticides accompanying biological activity, and so, notwithstanding their natural origin, they may as well have unknown effects on the environment and non-target organisms [23,24,25].

In Greece, lemon thyme is cultivated for culinary reasons, and after harvest some botanical material is freely available as waste, usually used for animal feed. We chose to use this material in an alternative way to control *Meloidogyne* sp for the first time, helping conserve human health and the environment by lessening the risk for water, air, soil, plants or animals in the frame of the European Waste Framework Directive [26]. We used lemon thyme culture residues powder (**P**) to prepare products to fight root knot nematodes but at the same time enhance plant growth and support soil microbes and free-living nematodes. The extraction techniques employed standard apparatus and the ratio of plant material to eluent was always 1/10 (*w*/*v*), as has usually been employed in the literature [27,28,29].

In particular, the scope of the study was to perform laboratory experiments so as (I) to calculate the paralysis activity of various *Thymus citriodorus* products on *Meloidogyne incognita* and *Meloidogyne javanica*; (II) to compare the arrest of the *M. javanica* biological cycle in tomato roots after soil treatment with the culture residues in the form of powder (**P**) at 1 g kg^−^^1^ of soil; and compare this activity against the contained in the powder essential oil (**EO**), hydrosol (**H**), water extract unfiltered (**WE**) and water extract filtered (**WEF**) used individually (all contained extracts yielded from 1 g plant powder and were incorporated at 1 kg of soil); (III) delineate the phytochemistry of the above extracts and discern amongst the ingredients for activity; (IV) to pinpoint the secondary beneficial effects of the various nematicidal lemon thyme products on the soil bacterial and fungal biomasses and free-living nematode abundances; and lastly, to (V) study the bio-fertilizing effect of **P** on tomato host plants after soil incorporation in a dose-response manner.

## 2. Results

### 2.1. Essential Oil (EO), Hydrosol (H) and Water Extract Filtered (WEF) Chemical Analysis

According to the chemical composition analyses, the EO and H yielded essential oil components but the WE did not (data not shown). More terpenes were identified in the EO and their yield was higher with respect to values in H. Specifically, a total of 33 and 5 compounds covering 99.66 and 53.32% of the total composition of EO and H were identified by gas chromatography/mass spectrometry (GC/MS) (Table 1). The major component was geraniol, yielding 47.08 and 44.06 % in EO and H, respectively.

### 2.2. Paralysis Effect of Lemon Thyme Essential Oil (EO), Hydrosol (H) and Water Extract Filtered (WEF) on the Plant Parasitic Nematodes M. Incognita and M. Javanica Second Stage Juveniles (J2)

The EC_50_ values established for *M. incognita* after immersion of J2 in test solutions of NEMguard for 1 and 2 days were 0.048 and 0.040 (% *v*/*v* of formulated product); while for *M javanica* the respective EC_50_ values were 0.063 and 0.1753 (% *v*/*v* of formulated product) (data not shown). This means that, to some extent, *M. javanica* regained activity after 2 days of J2 immersion in the test solutions. According to the paralysis bioassays, a dose-response relationship was evident for all three lemon thyme extracts and paralysis activity increased over time (Table 2). The best effective extract was the EO, followed by WEF and finally H, for *M. incognita* and *M. javanica*. Similar sensitivity of the paralysis effect was shown for both nematode species, as EC_50_ values were calculated at similar levels. In all cases, paralysis established after 2 days of exposure was not reversible since paralyzed J2s never regained motility.

### 2.3. Effect of Lemon Thyme Powder (P), Essential Oil (EO), Hydrosol (H), Water Extract (WE) and Water Extract Filtered (WEF) on the Biological Cycle of M. incognita

**Figure 1:**
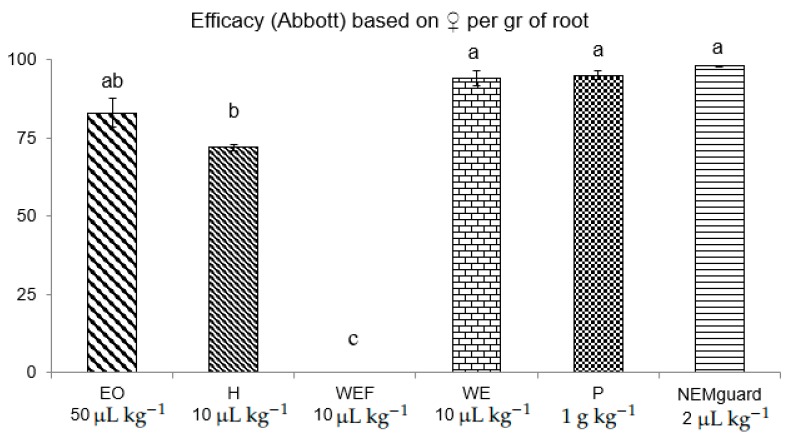
Nematicidal activity (± st. error) of different lemon thyme botanical products (EO: essential oil; H: hydrosol; WEF: water extract filtered; WE: water extract; P: powder and NEMguard) against *Meloidogyne incognita* based on female counts per gr of root, 45 days after treatment. Different letters above columns correspond to statistically significant differences between treatments.

When different lemon thyme products were used in the pot bioassay to assess the efficacy of 1% (w/w) P and the respective component EO, H and WE (filtered or not), the best effective treatment was the P and WE, which exhibited efficacy at the level of NEMguard (garlic extract) (Figure 1), namely 95, 94 and 98%, respectively. The EO followed and the H was last. Interestingly, the WEF did not exhibit any activity when applied in the pot experiment (Figure 1) and this might have to do with the stability of the extract inside the soil.

### 2.4. Effects of Lemon Thyme Powder (P), Essential Oil (EO), Hydrosol (H), Water Extract (WE) and Water Extract Filtered (WEF) on Soil Bacterial and Fungal Biomass, along with the Soil Microbial Feeding Nematodes

**Figure 2:**
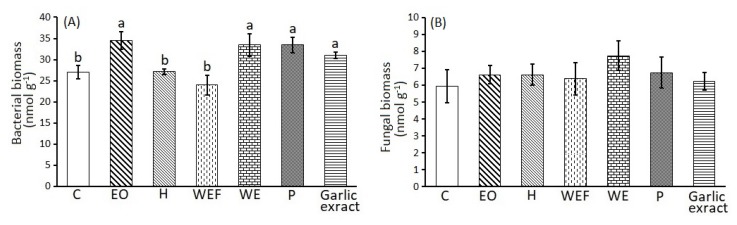
Mean values (± st. error) of soil bacterial and fungal biomass of the experimental plots (C: water control; EO: essential oil; H: hydrosol; WEF: water extract filtered; WE: water extract; P: powder and garlic extract), 45 days after treatment. Different letters above columns correspond to statistically significant differences between treatments, as revealed by one-way ANOVA (Tukey *p* < 0.05).

**Figure 3:**
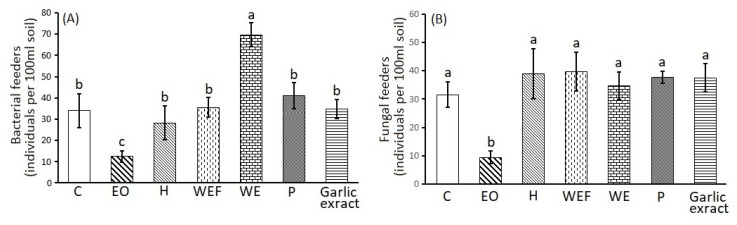
Mean values (± st. error) of soil bacterivorous and fungivorous free-living nematode abundances of the experimental plots (C: water control; EO: essential oil; H: hydrosol; WEF: water extract filtered; WE: water extract; P: powder and garlic extract), 45 days after treatment. Different letters above columns correspond to statistically significant differences between treatments, as revealed by one-way ANOVA (Tukey *p* < 0.05).

The results of the one-way ANOVA, regarding the effect of treatments on soil bacterial and fungal biomass, as well as on soil free-living bacterivorous and fungivorous nematodes, are given in Figure 2A,B and Figure 3A,B, respectively. Bacterial biomass was found to be higher in the EO, WE, P and garlic extract treatments and differed significantly compared to the control, WEF and H. On the contrary, the fungal biomass did not differ among the treatments. The EO treatment presented significantly lower abundances of bacterial and fungal feeding nematodes compared to the control. On the contrary, the WE treatment significantly increased the numbers of the bacterivorous nematodes, but not the fungivorous.

### 2.5. Soil Amendment with Lemon Thyme Powder (P) for Biofertilizing: A Dose-Response

**Figure 4:**
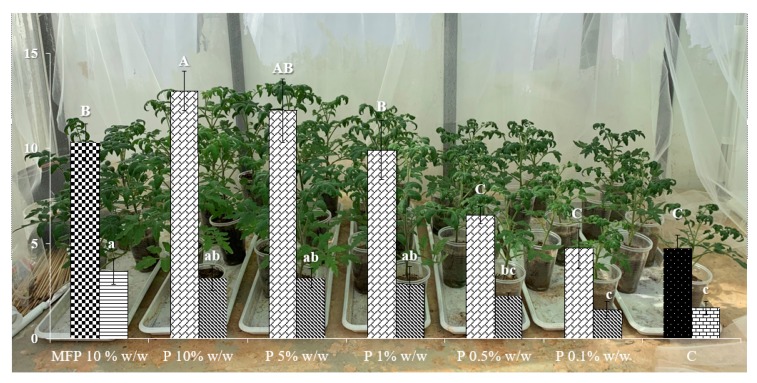
Weight increase of aerial parts and tomato roots after treatment with lemon thyme powder (P) at 0.1 to 10% against *Melia azedarach* powder 10% (MFP) and water control (C). Data are presented as means of five replicates with standard deviations. Means followed by the same letter are not significantly different according to Tukey’s test (*p* ≤ 0.05). Upper case letters correspond to statistical differences on fresh aerial parts weight. Lower case letters correspond to statistical differences on fresh roots weight.

A clear dose-response relationship was established considering fresh weights of both the aerial part and the root of tomatoes treated with increasing amounts of P as shown in Figure 4. No phytotoxicity was provoked on the tomato plants, even at the highest test concentration of 10% *w*/*w*. Interestingly, the biofertilizing effect was even greater at the test concentration of 10% *w*/*w* for lemon thyme if compared with *Melia azedarach* ripe fruit powder tested at the same test concentration. It has to be noted that *Melia azedarach* (Linnaeus) is a plant species proven to have significant biofertilizing properties based on our previous studies; thus, we chose to use it here as a reference for biofertilization.

## 3. Discussion

Our study showed that lemon thyme has great nematicidal potential against two plant parasitic nematodes and that at the same time it benefits soil microorganisms, free living nematodes and plant growth. Interestingly, the best effective lemon thyme products according to paralysis experiments were, in descending order of efficacy, the EOs followed by WEF and finally H, for *M. incognita* and *M. javanica* (Table 2). According to the chemical composition analyses (Table 1), the EOs, and to a much lesser extend H, are rich in constituent terpenes and in particular geraniol. In addition, other researchers have published similar composition analyses of lemon thyme EOs [15]. Indeed, the nematicidal potential of EOs and H can, to some extent, be attributed to geraniol content, since, according to our previous studies, geraniol showed paralysis activity on J2, and the EC_50_ value against *M. incognita* was calculated at 237 and 158 μg mL^−1^ after 24 and 28 h of immersion in test solutions [28]. Interestingly, geraniol was synergistic when in binary mixtures with *trans*-anethole and carvacrol [30], and carvacrol is indeed a chemical component of both EO and H according to Table 1; thus, it can synergistically attribute to the efficacy with geraniol. However, the efficacy of WEF exhibited in the paralysis bioassays (Table 2) was not manifested in the pot bioassays (Figure 1). On the contrary, if filtering is skipped and plant residues are included in the amendnment, then the WE intervenes with the parasites’ biological cycle (Figure 1). Interestingly, the unfiltered WE, together with P, were the most effective treatments at arresting the biological cycle inside the host roots according to female counts per gr of plant tissue. These two botanical products, representing a powder and a macerate, are the most chemically complex treatments used in the study and their nematicidal activity may be based not only on the supplied toxicity of plant secondary metabolites, e.g., terpenes, but also on their decomposition products, the changes in physical and chemical properties of the soil and the supply of biological parameters exhibiting activity [31,32,33,34]. On the other hand, the bacterial biomass was found to be higher in the EO, WE, and P treatments, but the EO treatment presented significantly lower abundances of bacterial and fungal feeding nematodes (Figure 2). On the contrary, the WE treatment significantly increased the numbers of the bacterivorous nematodes (Figure 3). This shows that WE and P are the most effective treatments against root knot nematodes and also the most beneficial according to soil parameters assessed herein. Lastly, the treatment of P furnished a clear dose-response biofertilizing effect (Figure 4), allowing applications even at higher concentration levels, without the fear of phytotoxicity, which may be useful in high nematode population levels. Interestingly, tomato growth was even greater at the test concentration of 10% *w*/*w* of lemon thyme, if one considers the *Melia azedarach* ripe fruit powder treatment tested at the same dose as a control according to our previous studies [35]. To the best of our knowledge, this is the first report on the comparative assessment of different botanical products, all originated from an aromatic species, against *M. incognita* and *M. javanica*. Additionally, we studied the ecotoxicity of these products and their effects on the tomato plants, along with their nematicidal activity. To date, many studies have shed light on the nematicidal activity of aromatic species (17–26) but no ecotoxicity data have been given at the nematicidal dose rates. In addition, in many cases the bioassays are performed *in vitro* so there are no phytotoxicity data. This is first step in the use of lemon thyme against RKN. We are now in the process of testing our hit lemon thyme products in open field studies.

## 4. Materials and Methods

### 4.1. Plant Material, Nematode Populations and Reagents

*Thymus citriodorus* was a kind offer from a local farmer (Etherikon, Greek Herbs) cultivating biological aromatic plants. The aerial parts were collected at the flowering stage and were dried in the absence of light at room temperature. Subsequently, they were sealed in paper bags and kept at room temperature, in the dark, until use. Τhe dried aerial plant parts were further processed into fine particle-powder (P), to be used thereafter for all extraction procedures and pot bioassays. Voucher specimens were taken to the Department of Ecology, School of Biology, Aristotle University of Thessaloniki, Greece, for species identification. Petroleum ether was of high-performance liquid chromatography grade. All chemical standards were obtained from Sigma-Aldrich (Milano, Italy).

*M. incognita* and *M. javanica* populations, originating from two single eggmasses of Greek origin were reared on tomato (*Solanum lycopersicum* Mill.) cv. Belladonna. Freshly hatched (24 h) second stage juveniles (J2) were extracted according to Hussey and Barker (1973) [36] from 60 day-old (d) infested roots, to be used for the bioassays.

### 4.2. Essential Oil (EO), Hydrosol (H) and Water Extract (WE and WEF) Production

The dried lemon thyme was subjected to water distillation using a Clevenger apparatus (Winzer) for 3 h at a ratio of 1/10 (*w*/*v*). That is, one hundred grams of aromatic plant was added to a 2000-mL glass flask with 1000 mL of distilled water. The EO obtained was dried over anhydrous Na_2_SO_4_ and stored in dark glass vial with Teflon-sealed caps at −20 ± 0.5 °C until use. The yield of EO determined on average over three replicates was 5 mL 100 g^−^^1^ of dry weight (data not shown). The remaining hydrosol in the glass flask was filtered and used fresh for bioassays while in part it was submitted to chemical analysis after 1/1 (*v*/*v*) fractionation in petroleum ether.

The water extract was produced by mixing the dried lemon thyme with distilled water at a ratio of 1/10 (*w*/*v*) and sonicated for 15 min. Thereafter, filtration took place in part of the extract through a Whatman no. 40 filter paper (Whatman International Ltd., Maidstone, England), while another extract, containing plant tissues, was kept. All samples were stored at −20 ± 0.5 °C until use for bioassays. Before chemical analysis all water containing extracts were subjected to 1/1 (*v*/*v*) fractionation in petroleum ether.

### 4.3. Chemical Analysis

Chromatographic separation and identification of the EOs main components was performed on a Trace GC Ultra gas chromatograph (Thermo Finnigan, San Jose, CA, USA) coupled with a Trace ISQ MS detector, a split-splitless injector, a Thermo Scientific™ TriPlus RSH autosampler (Rodano-Milan, Italy) and an Xcalibur MS platform. The EOs were diluted 1:100 (*v*/*v*) with hexane and one microliter of the diluted samples were injected with a split ratio of 50:1 on a 5% phenyl methylsiloxane fused silica capillary column (TR-5MS, 30 m length, 0.250 mm i.d., film thickness 0.25 μm). The injector and transfer line were at 220 °C and 220 °C respectively, the interface was at 250 °C, and the electron energy in the electron impact was 70 eV. Helium was the carrier gas at a constant flow rate of 1 mL/min. The GC oven temperature was programmed as follows: 70 °C (held for 5 min), then increased to 240 °C at a rate of 8 °C/min, and held at final temperature for 15 min. After 5 min of solvent delay, a mass range of m/z 50–600 was recorded. Mass spectrometry acquisition was carried out using the continuous electron impact ionization (EI) mode. The peak area integration and chromatogram visualization were performed using Xcalibur processing program. Peak identification and mass spectra tick evaluation was performed using the NIST11 database as well as by comparison of retention indices (RI) for alkanes C9–C24 with the ones reported by Adams 2007 [30] (Table 2).

### 4.4. Paralysis Effect of Lemon Thyme Essential Oil (EO), Hydrosol (H) and Water Extract Filtered (WEF) on the Plant Parasitic Nematodes M. incognita and M. javanica Second Stage Juveniles (J2)

The nematicidal activity of EO, H and WEF, in terms of J2 motility block, was studied, and the EC_50_ values were calculated. For each testing material a separate dose-response was established using 5 test concentrations ranging from 100 to 1000 μL L^−^^1^. Stock solutions of EO were made in ethanol, and working solutions were brought to volume with distilled water containing the polysorbate surfactant 20 (Tween-20). All other test solutions were prepared in water. Final concentrations of ethanol and Tween-20 in testing wells did not exceed 1 and 0.3% *v*/*v*, respectively. Distilled water and a carrier control, that is ethanol and Tween-20 at concentrations equivalent to those in the treatment wells, served as a control. Around fifteen J2 were used per treatment well in Cellstar 96-well plates (Greiner bio-one). The plates were covered and maintained in the dark at 28 °C. Border wells were used to check the vapor drift. Juveniles were observed with the aid of an inverted microscope (Euromex, The Netherlands) at 40 after 24 and 48 h and were ranked into two different categories: motile or paralyzed. After the last assessment (48 h), the nematodes were transferred into plain water, after washing in tap water through a 20 μm pore screen to remove the excess treatment substance(s), and they were assessed again after 24 h for motility regain. Nematodes that never regained activity were considered dead and the subsequent paralysis as nematidical activity. Paralysis treatments were replicated six times, and each experiment was performed twice.

### 4.5. Effect of Lemon Thyme Powder (P), Essential Oil (EO), Hydrosol (H), Water Extract (WE) and Water Extract Filtered (WEF) on the Biological Cycle of M. incognita

A clay loam soil with 1.3% organic matter and pH 7.8 was collected from a non-cultivated field of the University Farm. It was dispensed through a 3-mm sieve and was partially air dried overnight. Soil moisture (oven drying at 110 °C for 24 h) and maximum water holding capacity (MWHC) were measured according to Pantelelis et al., 2006 [37]. Next, the soil was mixed with sand at a ratio of 2:1 and the mix was separated into 6 plastic bags representing experimental treatments. Nematode inoculation was made with 2500 J2 kg^−^^1^ soil and an equal distribution of juveniles in soil was assured by mixing and incubation according to Ntalli et al., 2010 [28]. Based on preliminary trials, 1 g of **P** per kg of soil was nematicidal against *Meloidogyne incognita* at efficacy levels of 95%. To be able to understand if the activity of P is based in the contained EO, H, WEF or WE, we tested P against these fractions used individually, in a *M. javanica* control pot bioassay. In particular, the test concentration of each material per gr of soil was as follows: **P** 1 g kg^−^^1^ soil, **EO** 50 μL kg^−^^1^ soil, **H** 10 μL kg^−^^1^ soil, **WE** 10 μL kg^−^^1^ soil and **WEF** 10 μL kg^−^^1^ soil. We used 50 μL of EO kg^−^^1^ soil because that is the expected content of the EO (yield 5%) in 1 g of P. Likewise, we used 10 mL of H, WE and WEF because that is the amount of respective extracts that corresponds to 1 g P according to the extraction ratio of 1/10 (*w*/*v*). As a commercial control, we used the nematicide NEMguard SC (garlic extract) at the recommended dose for nematode control under field conditions (4 L ha^−^^1^ corresponding to 2 μL of formulated product per kg soil). Seven-week old tomato plants, cv. Belladonna, were transplanted into the treated soil and, at the end of the parasite cycle, inside the host roots, the efficacy assessments determined the number of *M. incognita* females per gram of root at 10× magnification control. The experiment was replicated once, and the treatments were always arranged in a completely randomised design with five replicates.

### 4.6. Effects of Lemon Thyme Powder (P), Essential Oil (EO), Hydrosol (H), Water Extract (WE) and Water Extract Filtered (WEF) on Soil Bacterial and Fungal Biomass along with the Soil Microbial Feeding Nematodes

On the 40th day, after the experiment described in Section 4.5, samples were taken to assess the Phospholipid Fatty Acid (PLFA) content of our soil samples as described in detail by Ntalli et al. (2018) [35]. The bacterial biomass was found as the sum of the 15:0, i-15:0, a-15:0, i-16:0, i-17:0, 16:1ω7c, 16:1ω9c, 16:1ω9t, cy17:0 and cy19:0 PLFA biomarkers, while the fungal biomass was equal to the 18:2ω9,12 one [38,39].

Soil free-living nematodes were extracted from 100 mL of each composite soil sample based on Cobb’s sieving and decanting method, as modified by S’Jacob and van Bezooijen (1984) [40]. We estimated the nematode abundance under a stereoscope, and then fixed them in 4% formaldehyde. From each sample, we randomly selected and identified 150 nematodes to the genus level [41]. Nematode genera were assigned to trophic groups according to [42]. In our plots, we found six bacterivorous genera (*Acrobeloides*, *Cervidellus*, *Rhabditis*, *Heterocephalobus*, *Chiloplacus*, *Panagrolaimus*) and three fungivorous genera (*Ditylenchus*, *Aphelenchus*, *Aphelenchoides*).

### 4.7. Soil Amending with Lemon Thyme Powder (P) for Biofertilizing: A Dose-Response

Appropriate amounts of P were mixed with the soil and sieved twice so as to achieve the concentrations of 0.1%, 0.5%, 1% and 5% *w*/*w*. A treatment with 10% *Melia azedarach* ripe powdered plain fruits served as control [35] since, according to our previous studies, it exhibits biofertilizing properties if incorporated in soil before transplanting the tomato plants. We also used a control treatment of water. The moisture content of the soil never exceeded 24% of the MWHC. After 24 h, the soil was used for transplanting 7-week old tomato plants, cv. Belladonna, into 200-g plastic pots and was maintained at 27 °C and 60% RH with a 16-h photoperiod. Each pot received 20 mL of water every 3 days, and the plants were uprooted and gently washed 40 days post experiment establishment. The weight of the aerial part along with the root was measured. The experiment was replicated once, and the treatments were always arranged in a completely randomized design with five replicates.

### 4.8. Statistics

Natural death/immobility was eliminated according to the Schneider Orelli formula [43]: corrected % = {(mortality% in treatment − mortality % in control)/(100 − mortality % in control)} × 100, and experiments were analyzed (ANOVA) and combined over time. Since ANOVA indicated no significant treatment by time interaction, means were averaged over experiments. Corrected percentages of paralyzed J2 were subjected to nonlinear regression analysis using the log-logistic equation proposed by Seefeldt et al. [44]: Y = C + (D − C)/{1 + exp[b (log(x) − log(EC_50_))]}, where C = the lower limit, D = the upper limit, b = the slope at the EC_50_, and EC_50_ = the test concentration required for 50% death/paralysis after removal of the control (natural death/paralysis). In the regression equation, the test concentration was the independent variable (x) and the paralyzed J2 (percentage increase over water control) was the dependent variable (y). The mean value of the six replicates per test concentration and immersion period was used to calculate the EC_50_ value.

Considering the pot bioassays, and since the ANOVAs indicated no significant treatment by time interaction (between runs of experiment), means were averaged over experiments. The data from the pot bioassays were expressed as a percentage decrease in the number of females per gram of root corrected according to the control (water), using the Abbott’s formula: corrected % = 100 × (1−(females number in treated plot/females number in control plot)). It was fitted in the log-logistic model, as for paralysis data, to estimate the concentration that caused a 50% decrease in females per gram of root (EC_50_ value).

We used one-way ANOVA to indicate the effect of different treatments on soil bacterial and fungal biomass as well as on free-living bacterivorous and fungivorous abundances. In all analyses, means were compared by Tukey’s test at *p* < 0.05.

## 5. Conclusions

There is a great need for eco-friendly nematode control tools. Efficacy, even at the regulatory level, is at times even less important than phyto- and ecotoxicity concerns. Consequently, both should be highlighted before suggesting tools and doses. This is a first report on the nematicidal potential of different aromatic products from *Thymus citriodorus* against *M. incognita* and *M. javanica* along with their side effects on soil microorganisms and free-living nematodes encompassing soil microcosms as well as nematode host plants. Lemon thyme powder and unfiltered water extract were the best active soil amendments that did not harm the microbes and saprophytic nematodes. This first acted as tomato bio-fertilizer in a dose response manner. Thus, our findings underline lemon thyme potential in an integrated pest-management program.

## Figures and Tables

**Table 1:** Chemical composition of essential oil (EO) and hydrosol (H), along with percentage contents of components.

No	Compound Name in Order of Elution ^a^	RI ^b^	EO ^c^	H ^c^
1	*a*-pinene	937	0.31	-
2	caphene	952	0.48	-
3	(-)-*β*-Pinene	978	0.09	-
4	1-octen-3-ol	980	0.25	-
5	3-octanone	985	1.72	-
6	3-octanol	993	1.06	-
7	*o*-cymene	1041	0.41	-
8	1,8 cineole	1032	0.74	-
9	*γ*-terpinene	1060	0.28	-
10	*p*-cymene	1090	-	1.19
11	*β*-linalool	1099	3.23	-
12	*β*-pinene oxide	1110	0.16	-
13	2,2-dimethylocta-3,4-dienal	1116	0.22	-
14	nerol oxide	1154	0.21	-
15	*cis*-verbenol	1144;	0.36	-
16	*endo*-borneol	1158;	2.44	-
17	*trans*-verbenol	1144	0.76	-
18	isopulegol	1167	0.61	-
19	*cis*-geraniol	1228	3.02	-
20	*β*-citral	1240	7.12	-
21	geraniol	1255	47.08	44.06
22	*α*-citral	1270	7.94	-
23	thymol	1290	2.93	2.22
24	carvacrol	1299	3.39	4.39
25	geranyl acetate	1380	4.05	-
26	(-)-*β*-bourbonene	1384	0.22	-
27	caryophyllene	1436	3.48	-
28	germacrene D	1480	0.61	-
29	*β*-bisabolene	1508	3.41	-
30	dihydroactinidiolide	1525	-	1.46
31	geraniol butyrate	1564	1.06	-
32	caryophyllene oxide	1581	1.54	-
33	butanoic acid	1596	0.48	-
Total identified compounds (%)			99.66	53.32
Number of identified compounds			33	5

^a^ Compounds are listed in order of elution from a TR-5MS, 30 m length,0.250 mm i.d., film thickness 0.25 μm. Identification by comparison of mass spectra with the respective data of NIST 11 library in continuous electron impact ionization (EI) mode, as well as retention indices as calculated according to Kovats, 1978, for alkanes C9 to C24 compared with those reported by Adams 2005 [30]. ^b^ Retention indices on the TR-5MS, 30 m length, 0.250 mm i.d., film thickness 0.25 μm. ^c^ Mean value of three determinations (three replicates) calculated from GC-MS areas; relative content <0.1%; (-) not detected.

**Table 2:** EC_50_ (% *w*/*v*) values of *M. incognita* and *M. javanica* calculated after immersion of J2s in test solutions of EO, WEF and H for 1 and 2 days.

	Days of Exposure	EC_50 (% *v*/*v*)_	Std. Error	CI_95_%
	EO			
*M. incognita*	1	0.091	0.087	0.095
	2	0.084	0.079	0.089
*M. javanica*	1	0.093	0.091	0.094
	2	0.092	0.090	0.094
	WEF			
*M. incognita*	1	2.860	2.470	3.251
	2	8.419	7.137	9.520
*M. javanica*	1	3.892	3.104	4.681
	2	6.197	4.401	7.993
	H			
*M. incognita*	1	38.950	26.704	51.196
	2	7.464	5.968	8.968
*M. javanica*	1	>50		
	2	4.353	3.703	5.003

## Abbreviations

Culture residues in the form of powder (P), essential oil (EO), hydrosol (H), water extract unfiltered (WE) and water extract filtered (WEF), gas chromatography/mass spectrometry (GC/MS).
